# A novel cyclodextrin glucanotransferase from an alkaliphile *Microbacterium terrae* KNR 9: purification and properties

**DOI:** 10.1007/s13205-016-0495-6

**Published:** 2016-08-13

**Authors:** Kiransinh N. Rajput, Kamlesh C. Patel, Ujjval B. Trivedi

**Affiliations:** 1 Department of Microbiology and Biotechnology, University School of Sciences, Gujarat University, Navrangpura, Ahmedabad, Gujarat 380 009 India; 2 Department of Microbiology, BRD School of Biosciences, Sardar Patel Maidan, Sardar Patel University, Satellite Campus, Bakrol, Vallabh Vidyanagar, Gujarat 388 120 India

**Keywords:** Cyclodextrin glucanotransferase, Alkaliphile, *Microbacterium terrae*, Starch adsorption, Purification

## Abstract

Cyclodextrin glucanotransferase (CGTase, EC. 2.1.1.19) produced using new alkaliphile *Microbacterium terrae* KNR 9 has been purified to homogeneity in a single step by the starch adsorption method. The specific activity of the purified CGTase was 45 U/mg compared to crude 0.9 U/mg. This resulted in a 50-fold purification of the enzyme with 33 % yield. The molecular weight of the purified enzyme was found to be 27.72 kDa as determined by SDS-PAGE. Non-denaturing gel electrophoresis and activity staining confirmed the presence of CGTase in crude and the ammonium sulfate precipitate fraction. The purified CGTase has a pI value of 4.2. The optimum pH of 6.0 and 60 °C temperature were found to be the best for CGTase activity. Purified CGTase showed 5.18 kcal/mol activation energy (Ea). The CGTase activity was increased in the presence of metal ions (5 mM): Ca^+2^ (130 %), Mg^+2^ (123 %), Mn^+2^ (119 %) and Co^+2^ (116 %). The enzyme activity was strongly inhibited in the presence of Hg^+2^ (0.0 %), Cu^+2^ (0.0 %) and Fe^+2^ (3.8 %). Inhibitor *N*-bromosuccinimide (5 mM) showed the highest 96 % inhibition of CGTase activity. SDS and triton X-100 among different detergents and surfactants (1.0 %, w/v) tested showed 92 % inhibition. Among the organic solvents checked for their effect on enzyme activity, 5 % (v/v) toluene resulted in 48 % increased activity. Polyethylene glycol-6000 showed a 26 % increase in the CGTase activity. The kinetic parameters *K*_m_ and *V*_max_ were 10 mg/ml and 146 µmol/mg min, respectively, for purified CGTase.

## Introduction

Amylases are of great significance for biotechnological and industrial applications and have approximately 25 % of the world enzyme market (Reddy et al. 2003). Cyclodextrin glucanotransferase is one of the important members of the α-amylase family 13 of glycosyl hydrolases which can degrade starch. This family of enzymes exhibits diversity in reaction specificities. Amylases generally hydrolyze glycosidic bonds in the starch molecules, but CGTases catalyze transglycosylation as a major reaction with hydrolysis being a minor activity (Van der Veen et al. 2000).

CGTases produce cyclodextrins (CDs) as a result of intramolecular transglycosylation (cyclization) reaction during the degradation of starch. Cyclodextrins are cyclic oligosaccharides commonly composed of six, seven or eight d-glucose units (α, β and γ-cyclodextrins, respectively) joined by α-(1, 4) glycosidic bonds (Szejtli 1998). Because of this unique property, CDs can form molecular inclusion complexes (host–guest complexes) with a wide range of solid, liquid and gaseous compounds and hence affect the stability, solubility, reactivity and bioavailability of a wide range of molecules. So, cyclodextrins have various applications in the field of medicine, food, pharmaceutical, analytical chemistry, agriculture and cosmetics (Hedges 1998). Though CDs have several possible industrial uses, their extensive applications are restricted due to their high prices and low yields (Singh et al. 2002). Hence, screening of novel sources for CGTases has a wide technical and practical impact on the enzymatic production of CDs (Biwer et al. 2002).

The bacillus group of bacteria, e.g., *Bacillus coagulans* (Akimaru et al. 1991), *Bacillus firmus* (Goel and Nene 1995), *Bacillus agaradhaerens* (Martins and Hatti-Kaul 2000), *Bacillus lentus* (Sabioni and Park 1992), *Bacillus ohbensis*, *Bacillus circulans, Bacillus macerans* (Yagi et al. 1986), *Bacillus alcalophilus* B-3101 (Abelyan et al. 2002) and *Bacillus pseudalcaliphilus* 20RF (Atanasovva et al. 2011) have been mainly reported for CGTase production. Apart from the Bacillus group, there are reports of CGTase production from other organisms like *Klebsiella pneumoniae* AS-22 (Gawande and Patkar 2001), *Brevibacterium sp.* 9605 (Mori et al. 1994), *Paenibacillus campinasensis* H69-3 (Alves-Prado et al. 2007), *Paenibacillus illinoisensis* ZY-08 (Lee et al. 2013) *Pyrococcus furiosus* DSM3638 (Lee et al. 2007), *Thermococcus* (Tachibana et al. 1999) and *Thermoactinomyces vulgaris* Tac-5354 (Abelyan et al. 2002). There are also reports of CGTase producing anaerobic bacteria like *Thermoanaerobacterium thermosulfurigenes* EM1 (Wind et al. 1995) and *Anaerobranca gottschalkii* (Thiemann et al. 2004). Bautista et al. (2012) reported the CGTase from halophilic archeon *Haloferax mediterranei*. Though CGTase production and characterization from a variety of aerobic mesophilic bacteria have been reported, there is a need of robust enzymes from other organisms considering the harsh conditions used in starch industries (Wind et al. 1995).

There are many reports on the purification of CGTases using different procedures like ultrafiltration, gel filtration, starch adsorption chromatography, hydrophobic interaction chromatography, ion exchange chromatography and affinity chromatography. We have isolated a new alkaliphile *Microbacterium terrae* KNR 9 for CGTase production (Rajput et al. 2016). In this study, we report the purification and properties of CGTase produced by *Mic. terrae* KNR 9.

## Materials and methods

### Materials

Cornstarch was purchased from Hi-media, Mumbai, India. Ammonium sulfate was bought from Qualigens India Ltd. Standard protein molecular weight markers were procured from Bangalore Genei (GeNei^TM^). All other chemicals used were of analytical grade.

### Organism and production medium

We have isolated the CGTase producing bacteria from fertile soil (Anand District, Gujarat, India) in our laboratory as described by Park et al. (1989). The highest enzyme-producing bacterial isolate was identified as *Microbacterium terrae* KNR 9 by IMTECH, Chandigarh, India, and deposited as *Microbacterium terrae* MTCC 8083 at the same institute. CGTase production was carried out in 100 ml medium containing 20 g/l soluble starch, 10 g/l yeast extract, 1.0 g/l K_2_HPO_4_, 0.2 g/l MgSO_4_·7H_2_O and 10 g/l Na_2_CO_3_ (autoclaved separately) in 250 ml flasks at 30 °C, 150 rpm on a rotary shaker for 72 h. After incubation, cells were removed by centrifugation and the supernatant used for enzyme purification.

### CGTase activity and protein estimation

CGTase activity was determined by the phenolphthalein assay method described by Goel and Nene (1995) with minor modification. 100 μl appropriately diluted purified enzyme was incubated with 1.0 ml of 50 mg soluble starch in sodium phosphate buffer (pH 6.0, 50 mM) at 60 °C for 30 min. The reaction was stopped by quickly cooling the tubes on ice. Four milliliters of working phenolphthalein solution was added and the tubes were vortexed. Absorbance of the mixture was immediately measured at 550 nm. The working phenolphthalein solution was prepared by adding 1 ml of phenolphthalein stock (4 mM in ethanol) to 100 ml of 125 mM Na_2_CO_3_ containing 4 % ethanol. The amount of β-CD produced was estimated from a standard curve of 0–100 μg/ml pure β-CD obtained by the same procedure. One unit was defined as the amount of enzyme that produced one μmole of β-CD per minute under assay conditions.

Protein estimation was carried out as described by Lowry et al. (1951). The standard protein calibration curve was prepared using pure bovine serum albumin (0–100 μg/ml).

### Ammonium sulfate fractionation

Cell-free supernatant was subjected to precipitation by the addition of (ammonium sulfate) (NH_4_)_2_SO_4_ powder to achieve 20 % saturation in an ice bath. Slow and gentle stirring of the mixture was performed for 2 h for better dissolution of ammonium sulfate to promote salting out of proteins. Centrifugation was carried at 9000*g* for 20 min at 4 °C and a pellet of protein obtained was checked for CGTase activity. Since the pellet did not show any CGTase activity, it was discarded. The supernatant was added with ammonium sulfate to achieve 80 % saturation and kept in an ice bath with gentle stirring for 2 h. The mixture was kept at 4 °C overnight to enhance the precipitation and stabilization of the enzyme. The resulting precipitates were separated from the supernatant by centrifugation at 9000*g* for 20 min at 4 °C and resuspended in 50 mM phosphate buffer, pH 6.0 and dialyzed against the same buffer at 8 °C for 24 h with three buffer changes. The CGTase activity and protein concentration were measured as mentioned above for the next purification steps.

### Purification of CGTase by starch adsorption

We purified the CGTase using the starch adsorption method described by Martins and Hatti-Kaul (2000) with minor modifications. For starch adsorption of CGTase, the crude enzyme was precipitated at 20 % ammonium sulfate saturation and the precipitated protein pellet obtained was discarded and the resultant supernatant of 20 % ammonium sulfate saturation containing soluble proteins subjected to cornstarch adsorption. In the other experiment, crude enzyme was added to plain 5 % (w/v) cornstarch without 20 % (w/v) ammonium sulfate to compare its effect on the adsorption of the enzyme.

To 20 ml crude enzyme, 20 % (w/v) ammonium sulfate was added and kept at 4 °C for 2 h on the stirrer. Then, it was centrifuged at 9000*g* and 4 °C for 20 min and the supernatant was carefully transferred to another flask discarding the pellet of protein precipitate. To this supernatant with 20 % (w/v) ammonium sulfate, 5 % (w/v) cornstarch was added and kept for 1 h at 8 °C with gentle stirring to allow enzyme adsorption on starch molecules. The mixture was centrifuged at 5000 rpm for 10 min to sediment the cornstarch with adsorbed proteins. The starch sediment obtained was washed twice with 10 ml of cold distilled water to remove unbound proteins. The adsorbed CGTase on the starch was eluted by incubating it with 5 ml of 1 mM β-CD in 50 mM phosphate buffer, pH 6.0, for 30 min at 37 °C on the stirrer. After that it was centrifuged at 9000*g* and 4 °C for 20 min to remove starch particles. The supernatant containing desorbed CGTase was carefully transferred to another tube. The elution was repeated again with 2.0 ml of the elution buffer. Elutes were pooled and dialyzed against 50 mM phosphate buffer, pH 6.0, at 8 °C for 24 h with three buffer changes. In the other experiment, 20 ml crude enzyme was mixed with 5 % (w/v) cornstarch only and incubated at 8 °C for 1 h with gentle stirring. After that, 5 ml of 1 mM β-CD in 50 mM phosphate buffer, pH 6.0, was added and kept for 30 min at 37 °C on the stirrer to recover adsorbed enzyme.

### Characterization of the purified CGTase

#### Determination of molecular weight

The molecular weight of the purified protein (CGTase) was estimated by sodium dodecyl sulfate–polyacrylamide gel electrophoresis (SDS-PAGE). It was performed on a vertical slab gel using 10.0 % (w/v) acrylamide gel at a constant voltage of 125 V. Standard protein molecular weight markers (GeNei^TM^) used were phosphorylase B (M_r_ 97,400 Da); bovine serum albumin (M_r_ 66,000 Da); ovalbumin (M_r_ 43,000 Da); carbonic anhydrase (M_r_ 29,000 Da); soybean trypsin inhibitor (M_r_ 20,100 Da); and lysozyme (M_r_ 14,300 Da). The gel was stained by the silver staining method. The molecular weight of the enzyme protein was determined using an Alpha DigiDoc^Tm^ (Alpha Innotech, California, USA) with the Alpha DigiDoc^®^RT software.

#### Non-denaturing gel electrophoresis and activity staining

Native polyacrylamide gel electrophoresis was performed in 8 % gel at a constant voltage of 100 V till the dye front reached the end of the resolving gel. Crude and dialyzed enzyme samples of ammonium sulfate precipitation were analyzed to check the presence of enzyme protein. After completion of the electrophoretic run, the gel was rinsed twice with 50 mM phosphate buffer (pH 6.0).

Activity staining of the gel was done according to Pakzad et al. (2005) with minor modifications. The starch indicator gel was prepared before completion of an electrophoretic run using 0.5 g soluble starch and 0.4 g agar in 20 ml of 50 mM phosphate buffer, pH 6.0, under boiling condition. After that, 0.5 ml of 0.4 g % phenolphthalein was added to this mixture and it was poured in a Petri plate upon cooling to 50 °C. The native gel was carefully transferred by placing it on the starch indicator gel and incubated at 50 °C for 10 min. During incubation, enough care was taken to ensure that the gel remains flooded with a phosphate buffer (50 mM, pH 6.0). After incubation, the native gel was removed carefully and the starch indicator gel flooded with a 0.1 g % sodium carbonate solution until the background of the gel turned pink. The presence of a colorless band in the starch indicator gel confirmed CGTase activity of the separated protein.

After visualization of CGTase activity, the native PAGE gel was subjected to a silver staining method and compared with the starch indicator gel.

#### Isoelectric focusing

Isoelectric focusing was carried out at 20 °C in the Protean IEF cell (BioRad, USA) according to the manufacturer’s instructions. The isoelectric point of the purified CGTase was analyzed using immobilized pH gradient (IPG) strips (BioRad, USA) with a pH range of 3.0–10.0. Active rehydration of the IPG strip was carried out by keeping it in the solution containing purified CGTase and rehydration buffer at 50 V for 12 h. The rehydration buffer comprised 8 M urea, 2 % CHAPS, 50 mM dithiothreitol (DTT), 0.2 % carrier ampholytes and 0.001 % Bromophenol Blue. After rehydration of the IPG strip, focusing was carried out on Protean IEF cell (BioRad, USA) initially at 250 V for 15 min, 10,000 V for the next 3 h and the final focusing was done till 40,000 Vh was achieved. The enzyme protein was visualized by silver staining of the IPG strip.

#### Effect of pH on CGTase activity

The effect of pH on CGTase activity was checked using the phenolphthalein assay method as described earlier. The optimum pH of the purified CGTase enzyme was determined by replacing 50 mM phosphate buffer (pH 6.0) with the followings buffers: 50 mM sodium acetate buffer (pH 4.0, 5.0 and 5.5), 50 mM sodium phosphate buffer (pH 6.0, 6.5 and 7.0), 50 mM Tris–HCl (pH 8.0) and 50 mM glycine–NaOH buffer (pH 9.0, 10.0 and 10.5). Considering the enzyme activity at the best pH as 100 %, a pH profile of the relative activity versus pH was plotted.

#### Effect of temperature on CGTase activity

The effect of temperature on enzyme activity was tested at different temperatures. The optimum temperature for CGTase activity was determined by incubating the reaction mixture of CGTase assay at different temperatures, ranging from 30 to 70 °C in 50 mM phosphate buffer (pH 6.0) for 20 min. Considering the enzyme activity at the best temperature as 100 %, a temperature profile of the relative activity versus temperature was plotted.

#### Effect of metal ions on CGTase activity

Effect of different metal salts, namely CaCl_2_, MgSO_4_, FeSO_4_, ZnSO_4_, CuSO_4_, MgCl_2_, MnCl_2_, ZnCl_2_, CoCl_2_, HgCl_2_, NiCl_2_, K_2_Cr_2_O_7_ and AgNO_3_ on CGTase enzyme activity was determined. Appropriately diluted 100 µl of enzyme was mixed with metals (5 mM final concentration) and incubated at 25 °C for 10 min. The residual activity of the enzyme was determined.

#### Effect of inhibitors on CGTase activity

The effect of different inhibitors, namely *p*-chloromercuribenzoic acid (*p*-CMB), phenylmethylsulfonyl fluoride (PMSF), N-bromosuccinimide (NBS), dithiothreitol (DTT), ethylenediamine tetraacetate (EDTA) and β-mercaptoethanol (β-ME), on CGTase activity was checked. Appropriately diluted 100 µl of an enzyme was mixed with the respective inhibitors (5.0 mM final concentration) and incubated at 25 °C for 10 min. Residual enzyme activity was determined.

#### Effect of detergents on CGTase activity

Effect of various detergents (1.0 %, w/v), namely, cetyl trimethyl ammonium bromide (C-TAB), sodium dodecyl sulfate (SDS), Tween 40, Tween 80 and Triton X-100 on CGTase activity was checked. The approximately diluted 100 µl enzyme was mixed with the detergents and incubated at 25 °C for 10 min. After that, the residual enzyme activity was determined by the CGTase assay.

#### Effect of organic solvents on CGTase activity

Effect of various organic solvents, viz. acetone, ethanol, isopropanol, hexane, cyclohexane, toluene, iso-octane and dodecane, on CGTase activity was checked. Organic solvents (5 %, v/v) were added to the tubes containing substrate (0.5 g % soluble starch) and approximately diluted 100 µl enzyme. The reaction mixture was incubated at 25 °C for 10 min followed by the CGTase assay method.

#### Effect of polyols on CGTase activity

The effect of different polyols, viz. glycerol, mannitol, sorbitol and polyethylene glycol, on CGTase activity was determined. Different polyols (0.5 M final concentration) were mixed with soluble starch (0.5 g % final concentration) and then appropriately diluted 100 µl enzyme was added to it. The reaction mixtures were incubated at 60 °C for 20 min followed by the CGTase assay method.

#### Kinetic parameters

*K*_m_ and *V*_max_ values for the purified CGTase enzyme were determined. Fixed 0.27 µg of purified CGTase protein was incubated with different soluble starch concentrations ranging from 0.5 to 5.0 mg/ml in 1.0 ml of 50 mM phosphate buffer, pH 6.0 at 60 °C for 4 min. The values of *K*_m_ and *V*_max_ were determined from a Lineweaver–Burk plot.

## Results and discussion

### Concentration by ammonium sulfate precipitation

CGTase production was performed as mentioned earlier and the cell-free supernatant, i.e., crude enzyme was used for further purification. This crude CGTase enzyme was first concentrated by precipitation following ammonium sulfate fractionation. The protein pellet of 0–20 % showed no CGTase activity. The 20–80 % salt saturation concentrated protein pellet showed 9.84 U/mg of specific CGTase activity with 72 % yield (Table 1).

**Table 1:** Results of purification of CGTase from *Mic. terrae* KNR 9

Procedure	Activity (U/ml)	Protein (mg/ml)	Specific activity (U/mg)	Purification fold	Yield (%)
Crude enzyme	4.71	5.23	0.90	1	100
Ammonium sulfate precipitation (20–80 %)	23.52	2.39	9.84	10.9	72
Starch adsorption	6.06	0.13	45.22	50	33

### Purification of CGTase by starch adsorption

The CGTase from *Mic. terrae* KNR 9 was purified to homogeneity by adsorption onto cornstarch followed by elution with buffer containing β-cyclodextrin (Martins and Hatti-Kaul 2000). The precipitation and adsorption of CGTase were promoted in the presence of 20 % ammonium sulfate. In an experiment where plain cornstarch was used directly for the adsorption of the crude enzyme, only 9.31 % yield was obtained. In the other method, crude enzyme was precipitated at 20 % ammonium sulfate saturation and the precipitated protein pellet obtained was discarded. The resultant supernatant of 20 % ammonium sulfate saturation containing soluble proteins was subjected to cornstarch adsorption. By this method, CGTase from *Mic. terrae* KNR 9 could be purified to homogeneity in a single step with 45.22 U/mg specific activity, 50-fold purification and 33 % yield (Table 1).

Martins and Hatti-Kaul (2000) reported purification of CGTase from *B. agaradhaerens* LC-3C using the starch adsorption method with 50 % yield and 43-fold purification. Rosso et al. (2005) described the CGTase purification from *B. circulans* using the starch adsorption method. Vassileva et al. (2007) obtained purified CGTase with 87 % enzyme yield and 1.3-fold purification.

### Characterization of CGTase from *Mic. terrae* KNR 9

#### Molecular weight determination

**Fig. 1:**
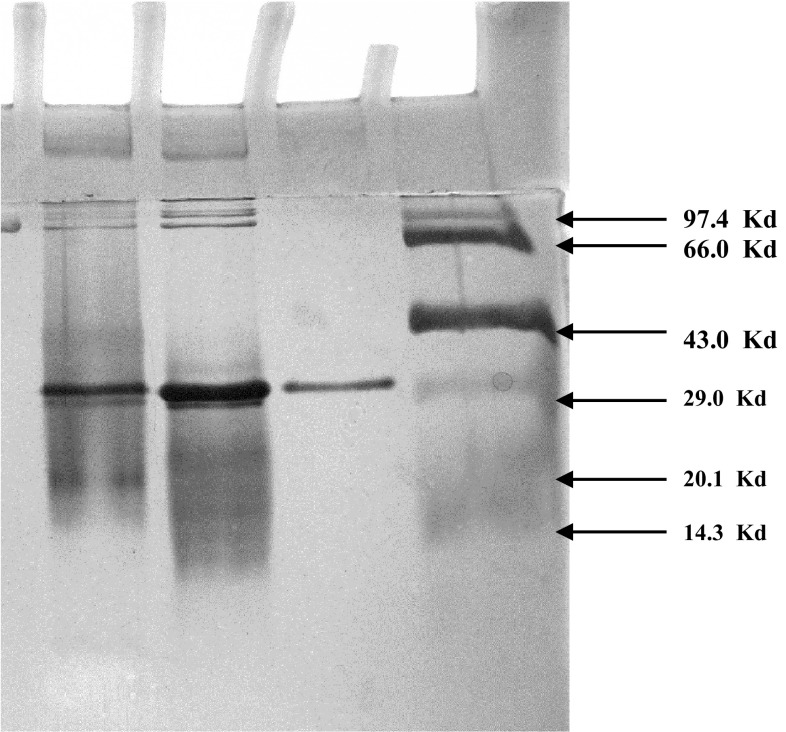
SDS-PAGE of the purified CGTase. *Lane 1* crude CGTase, *Lane 2* ammonium sulfate-concentrated enzyme, *Lane 3* purified CGTase by starch adsorption, *Lane 4* molecular weight markers

Sodium dodecyl sulfate–polyacrylamide gel electrophoresis (SDS-PAGE) is a widely used method for identification, screening and assessment of homogeneity of the purified protein fractions. The purified CGTase exhibited a single protein band on the 10 % polyacrylamide gel stained by silver indicating that it was a monomer protein (Fig. 1). The molecular weight of the purified enzyme was assessed by electrophoretic mobility of proteins in the denaturing SDS-PAGE gel. The molecular weight of CGTase from *Mic. terrae* KNR 9 was 27.72 kDa compared to the distance traveled by individual standard molecular weight markers using the Alpha DigiDoc^®^RT software. To the best of our knowledge, this is the smallest molecular weight CGTase reported so far.

The other CGTases reported having lower molecular weights are *B. coagulans*, 36 kDa (Akimaru et al. 1991), and *B. lentus*, 33 kDa (Sabioni and Park 1992). The majority of purified CGTases reported in the literature have a molecular weight between 65 and 80 kDa, e.g., *Bacillus pseudalcaliphilus* 20RF, 70 kDa (Atanasovva et al. 2011), *Paenibacillus campinasensis* strain H69-3, 70 kDa (Alves-Prado et al. 2007), *Paenibacillus illinoisensis* ZY-08, 74 kDa (Lee et al. 2013), *B. stearothermophilus* ET1, 66.88 kDa, (Chung et al. 1998), *Bacillus firmus*, 78 kDa (Gawande et al. 1999), *Bacillus pseudalcaliphilus* 8SB, 71 kDa, (Kitayska et al. 2011) and *Bacillus megaterium* 73.4 kDa (Pishtiyski et al. 2008). On the other hand, CGTase from *B. agaradhaerens* LC-3C has a higher molecular weight of 110 kDa (Martins and Hatti-Kaul 2000). Abelyan et al. (2002) noticed that CGTases of alkaliphilic strains had higher molecular weight. In contrast to this, CGTase from new alkaliphile *Mic. terrae* KNR 9 has a lower molecular weight of 27.72 kDa.

#### Non-denaturing gel electrophoresis and activity staining

**Fig. 2:**
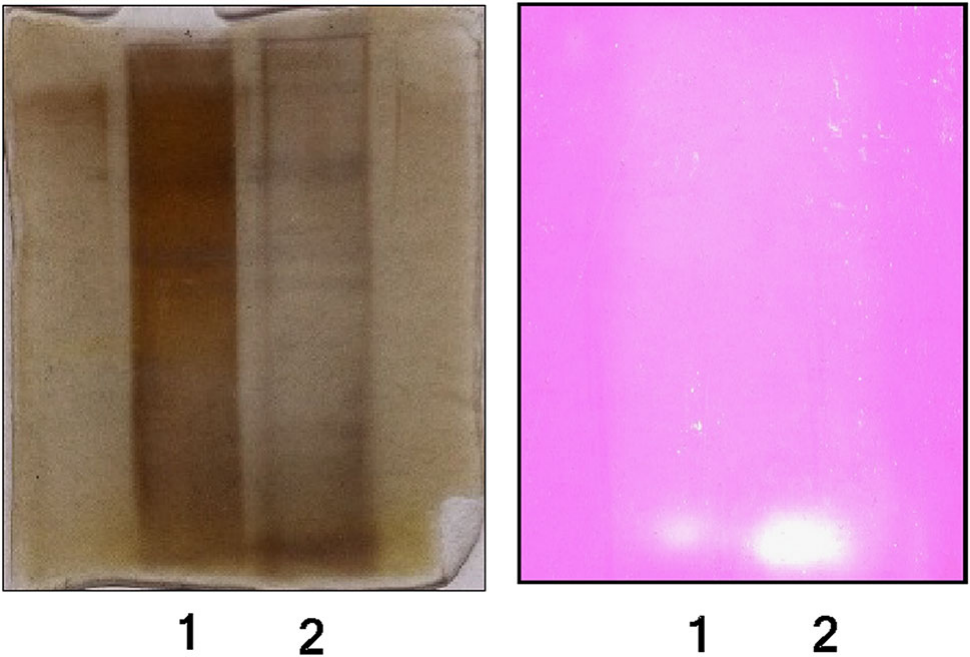
Native PAGE and activity staining. *Lane 1* crude enzyme, *Lane 2* ammonium sulfate-concentrated enzyme

The presence of CGTase was confirmed by non-denaturing gel electrophoresis followed by activity staining as described earlier. Two colorless bands appeared upon addition of 0.1 % (w/v) sodium carbonate in the pink-colored starch indicator gel indicating CGTase activity (Fig. 2). The corresponding two bands were observed in the non-denaturing gel after silver staining in crude and ammonium sulfate-concentrated enzyme lane. CGTase is an enzyme of the α-amylase family and shows starch degradation as like other amylases and can be detected by the addition of iodine solution on starch gel after native PAGE (Alves-Prado et al. 2007; Thiemann et al. 2004). However, it indicates starch hydrolysis only and cannot discriminate between two amylolytic enzymes. Activity staining with this specific phenolphthalein starch indicator gel method reveals only bands of CGTase activity (Pakzad et al. 2005).

#### Isoelectric focusing

The CGTase from *Mic. terrae* KNR 9 showed isoelectric pH (*pI*) of 4.2. Mori et al. (1994) and Yagi et al. (1986) also reported the acidic pI of 2.8 and <4.0 for CGTase from *Brevibacterium* sp. 9605 and *B. ohbensis*, respectively. The other reported *pI*s for CGTases are *Pae. campinasensis* H69-3, 5.3 (Alves-Prado et al. 2007), *B. agaradhaerens* LC-3C, 6.9 (Martins and Hatti-Kaul 2002), *K. pneumoniae* AS-22, 7.3 (Gawande and Patkar 2001), *B. circulans* IFO 3329, 8.8 (Yagi et al. 1986) and *Bacillus* sp. G1, 8.8 (Sian et al. 2005).

#### Effect of pH on CGTase activity

**Fig. 3:**
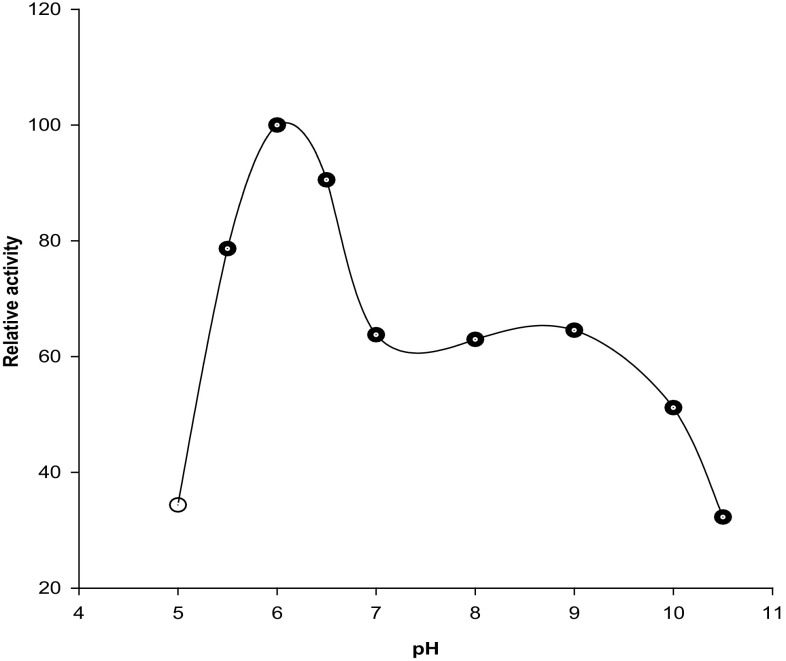
Effect of pH on CGTase activity

Enzyme activity was measured in different buffers between pH 4.0 to 10.5 at 60 °C using the CGTase assay method. The best enzyme activity was observed at 6.0 pH (50 mM phosphate buffer) and so it is the optimum pH for the purified enzyme (Fig. 3). The optimal pH value obtained is in accordance with other reported pH values in the literature. The CGTases from *B. circulans* ATCC 21783 (Vassileva et al. 2007), *B. stearothermophilus* ET1 (Chung et al. 1998), *Bacillus* sp. G1 (Sian et al. 2005), *B. firmus* No. 37 (Matioli et al. 2001) and *B. firmus* (Moriwaki et al. 2007) have shown the highest enzyme activity at 6.0 pH.

The CGTase from *Mic. terrae* KNR 9 showed no activity at pH ≤4.0 and ≥10.5. Extreme pH values were not found suitable for CGTase activity. It needs near-neutral pH for its highest activity. However, CGTase from *Mic. terrae* KNR 9 showed another broad peak of 60 % relative activity at 8.0–9.0 pH (Fig. 3). The other reported CGTases having near-neutral optimum pH are *B. coagulans* (Akimaru et al. 1991), *K. pneumoniae* AS-22 (Gawande and Patkar 2001), *Pae. campinasensis* H69-3 (Alves-Prado et al. 2007), *Pae. illinoisensis* ZY-08 (Lee et al. 2013), *Thermococcus* sp. (Tachibana et al. 1999), *B. pseudalcaliphilus* 8SB (Kitayska et al. 2011), *B. megaterium* (Pishtiyski et al. 2008), *B. firmus* (Yim et al. 1997) and *B. lehensis* (Elbaz et al. 2015).

#### Effect of temperature on CGTase activity

**Fig. 4:**
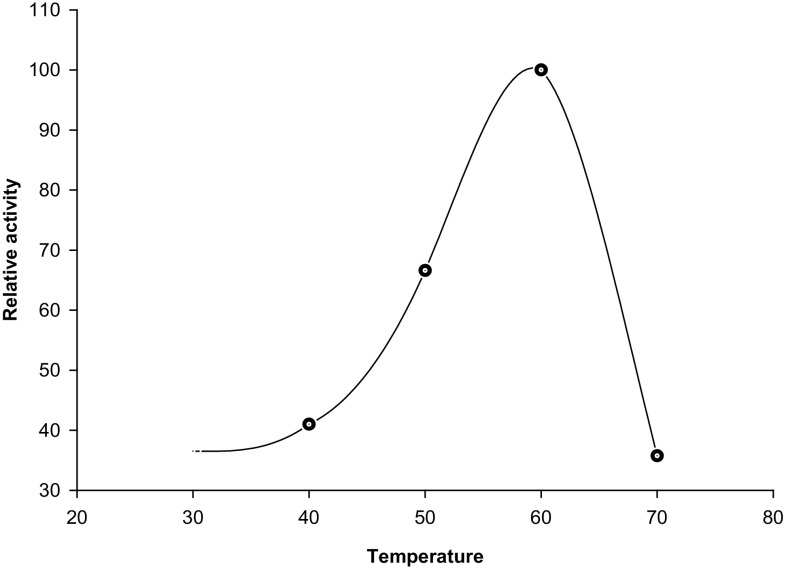
Effect of temperature on CGTase activity

The CGTase activity was determined at different temperatures ranging from 30 to 70 °C. The temperature of 60 °C showed the highest activity for the purified CGTase from *Mic. terrae* KNR 9 (Fig. 4). Similarly, a temperature of 60 °C was found to be optimum for CGTases of *B. megaterium* (Pishtiyski et al. 2008), *B. circulans* E192 (Bovetto et al. 1992) and *B. lehensis* (Elbaz et al. 2015). Most of the CGTases reported have optimum temperature between 50 and 65 °C for their activity (Atanasovva et al. 2011; Lee et al. 2007; Martins and Hatti-Kaul 2002; Matioli et al. 2001; Vassileva et al. 2007; Yim et al. 1997).

The higher optimum temperature of 75 °C has been noted for CGTases from *B. alcalophilus* B-3101 and *Thermoactinomyces vulgaris* Tac-5354 (Alves-Prado et al. 2007). There are reports of thermostable CGTases from *B. stearothermophilus* at 80 °C (Chung et al. 1998) and *Thermoanaerobacter thermosulfurigenes* EM1 at 80–85 °C (Wind et al. 1995). In contrast to this, Lee et al. (2013) noticed 40 °C as the best for CGTase activity of *Pae. illinoisensis* ZY-08.

#### Activation energy of CGTase from *Mic. terrae* KNR 9

**Fig. 5:**
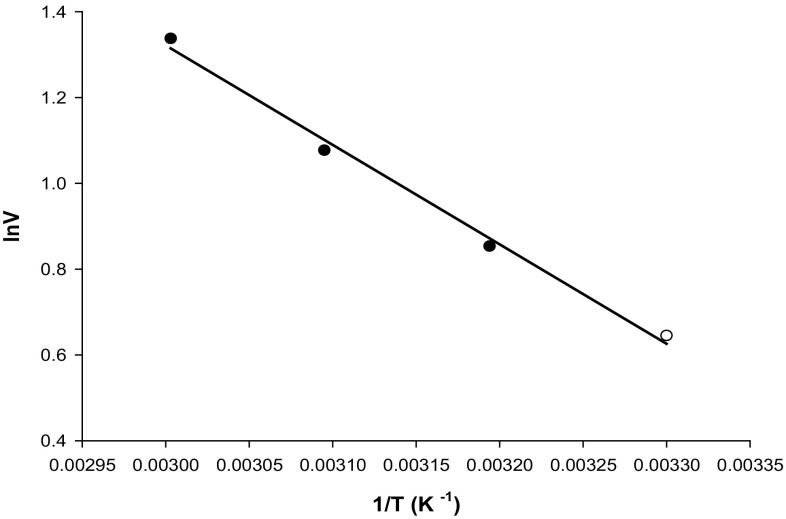
Arrhenius plot for activation energy

The effect of temperature on an enzymatic reaction can be analyzed through the Arrhenius equation by plotting the velocity (log_10_V) against the inverse of temperature (1/*T* expressed in K^−1^) (Fig. 5). For purified CGTase, the activation energy (Ea) determined was 5.18 kcal/mol. Reported *E*a for other different CGTases are *B. agaradhaerens* LC-3C, 17 kcal/mol (Martins and Hatti-Kaul 2002), *B. firmus* no. 37, 7.5 kcal/mol (Matioli et al. 2001) and *B. firmus*, 9.4 kcal/mol (Moriwaki et al. 2007).

#### Effect of metal ions on CGTase activity

To check the effect of metal ions on purified CGTase, the enzyme was incubated with some metal salts. Purified CGTase was added to metal salts 5.0 mM, in 50 mM phosphate buffer, pH 6.0 at 25 °C for 10 min and then the residual activity was determined. The results of CGTase activity are summarized in Table 2. The metal ions Hg^+2^ and Cu^+2^ completely inhibited the CGTase of *Mic. terrae* KNR 9, showing no residual activity (0.0 %). The purified CGTase was also strongly suppressed by Fe^+2^ and Ag^+3^ giving 3.84 % and 14.10 % residual activity, respectively. The CGTase was slightly inhibited in the presence of Zn^+2^ and Cr^+3^ showing about 90 % residual activity. No effect of Ni^+2^ was observed on CGTase activity. The CGTase activity was slightly enhanced in the presence of Ca^+2^, Mg^+2^, Co^+2^ and Mn^+2^.

**Table 2:** Effect of metal ions on CGTase activity

Metal salts	Residual activity (%)
Control (none)	100.00
HgCl_2_	0.00
CuSO_4_	0.00
FeSO_4_	3.84
AgNO_3_	14.10
ZnCl_2_	87.18
ZnSO_4_	91.03
K_2_Cr_2_O_7_	92.05
NiCl_2_	102.56
MgCl_2_	111.53
CoCl_2_	116.66
MnCl_2_	119.23
MgSO_4_	123.08
CaCl_2_	130.76

A strong inhibition of CGTase activity with Hg^+2^ has been reported by Akimaru et al. (1991) and Martins and Hatti-Kaul (2002). There are reports of significant inhibitory effect of Cu^+2^ on CGTases of various bacteria (Akimaru et al. 1991; Mori et al. 1994; Kitayska et al. 2011; Martins and Hatti-Kaul 2002; Sian et al. 2005; Yim et al. 1997). Similar inhibitory effects of Fe^+2^ were reported for CGTase from *Bacillus* sp. AL-6 (Fujita et al. 1990) and *B. agaradhaerens* LC-3C (Martins and Hatti-Kaul 2002). Similar to CGTase from *Mic. terrae* KNR 9, CGTase from *B. megaterium* (Pishtiyski et al. 2008), *Bacillus* sp. G1 (Sian et al. 2005) and *Bacillus* sp. AL-6 (Fujita et al. 1990) showed inhibition with Zn^+2^. Most of the reported CGTases showed either enhanced CGTase activity and stability or retained the activity in the presence of Ca^+2^ (Akimaru et al. 1991; Mori et al. 1994; Lee et al. 2013; Kitayska et al. 2011; Martins and Hatti-Kaul 2002; Sian et al. 2005; Yim et al. 1997). The improvement in the activity could be related to the interactions of Ca^+2^ ions with the amino acids near the active site that are conserved among the α-amylase family enzymes (Martins and Hatti-Kaul 2002).

#### Effect of inhibitors on CGTase activity

The effect of different known enzyme inhibitors, viz. ethylenediamine tetraacetate (EDTA), phenylmethylsulfonylfluoride (PMSF), dithioerythritol (DTT), β-mercapto-ethanol (β-ME), N- bromosuccinimide (NBS) and *p*-chloromercuribenzoate (*p*-CMB), on the CGTase activity was checked (Table 3).

**Table 3:** Effect of inhibitors on CGTase activity

Inhibitors	Residual activity (%)
Control (none)	100.00
*N*-Bromosuccinimide	3.87
*p*-Chloromercuribenzoic acid (*p*-CMB)	60.00
EDTA	79.35
PMSF	92.25
Dithiothreitol (DTT)	97.41

N-Bromosuccinimide showed 96 % inhibition of the CGTase activity. It is known to oxidize the tryptophan residues present in the enzyme and thereby inhibit the enzyme activity (Spende et al. 1966). Hence, it can be stated that the tryptophan and related amino acids might be present in the active site of purified CGTase and have a crucial role in enzyme action. Similar results were reported with CGTase from *B. agaradhaerens* LC-3C (Martins and Hatti-Kaul 2002). Close to 40 % inhibition of enzyme activity was observed with p-CMB, indicating the presence of the sulfhydryl groups on the active site of the enzyme. None of the other inhibitors tested, i.e., EDTA, PMSF, DTT and β-ME, showed significant inhibition of CGTase activity. Similar results for the effect of inhibitors for CGTases from other sources have been reported (Akimaru et al. 1991; Fujita et al. 1990; Martins and Hatti-Kaul 2002; Yim et al. 1997).

#### Effect of detergents on CGTase activity

The effect of several detergents/surfactants on CGTase activity is summarized in Table 4. The highest inhibition of 92 % CGTase activity was observed with a nonionic detergent Triton X-100. Anionic surfactants SDS and CTAB also showed higher inhibition of 91 and 75 %, respectively. Nonionic surfactants Tween-40 and Tween-80 revealed about 30 % inhibition of enzyme activity.

**Table 4:** Effect of detergents/surfactants on CGTase activity

Detergents/surfactants	Residual activity (%)
Control (none)	100.00
Triton X-00	8.04
SDS	8.72
C-TAB	24.16
Tween 40	69.12
Tween 80	71.14

#### Effect of organic solvents on CGTase activity

The effect of various organic solvents, viz. acetone, ethanol, isopropanol, hexane, cyclohexane, toluene, iso-octane and dodecane, on cyclodextrin production by CGTase is represented in Table 5. Among the tested organic solvents, toluene, dodecane, hexane, cyclohexane and iso-octane increased the cyclodextrin yield, whereas acetone and isopropanol showed decreased cyclodextrin production. The addition of toluene to the reaction mixture resulted in the highest 48 % increase of CGTase activity, followed by dodecane, hexane, cyclohexane and iso-octane.

**Table 5:** Effect of organic solvents on CGTase activity

Organic solvents	Residual activity (%)
Control (none)	100.00
Isopropanol	91.36
Acetone	94.96
Ethanol	103.59
Iso-octane	116.54
Cyclohexane	123.07
Hexane	123.84
Dodecane	131.65
Toluene	147.48

Abelyan et al. (2002) observed increased β-CD yield using various organic solvents with toluene showing the highest impact. Blackwood and Bucke (2000) reported the increase in cyclodextrin yield with CGTases from *Thermoanaerobacter* sp. and *B. circulans* 251 using different solvents. Qi et al. (2007) noticed the effect of ethanol on the yield of large-ring CDs using CGTase from *Bacillus* sp. BT3-2. Polar organic solvents can form inclusion complexes with cyclodextrins and may result in conformational changes in the cyclodextrin structure which can decrease product inhibition.

#### Effect of polyols on CGTase activity

The effect of different polyols at 0.5 M final concentration was determined on CGTase activity and the results are summarized in Table 6. Among the polyols tested, only PEG-6000 showed positive effect on CGTase with 26 % increased activity. Rest of the tested polyols have no effect or slightly inhibitory effect on CGTase activity.

**Table 6:** Effect of polyols on CGTase activity

Polyols	Residual activity (%)
Control (none)	100.00
Glycerol	80.40
Mannitol	85.13
Sorbitol	87.16
Polyethylene glycol (PEG-400)	96.62
Polyethylene glycol (PEG-6000)	126.70

Martins and Hatti-Kaul (2002) noticed the increased activity with sorbitol and PEG-3000. Delbourg et al. (1993) reported the improved activity in the presence of glycerol, mannitol and PEG-200, but decreased activity with xylitol and sorbitol. Addition of polyethylene glycol and polypropylene glycol increased the CD production by CGTase of *B. ohbensis* (Hayashida and Kawakami 1992). Yoon and Robyt (2005) have reported 20 % increased activity with PEG-1500 for CGTase from *B. macerans*. Cyclodextrin production may be influenced by factors causing changes in the substrate/enzyme or additive/active site interactions, or even with changes in the microenvironment of the enzyme (e.g., reduction of water activity) induced by the additive (Martins and Hatti-Kaul 2002).

#### Kinetic parameters

**Fig. 6:**
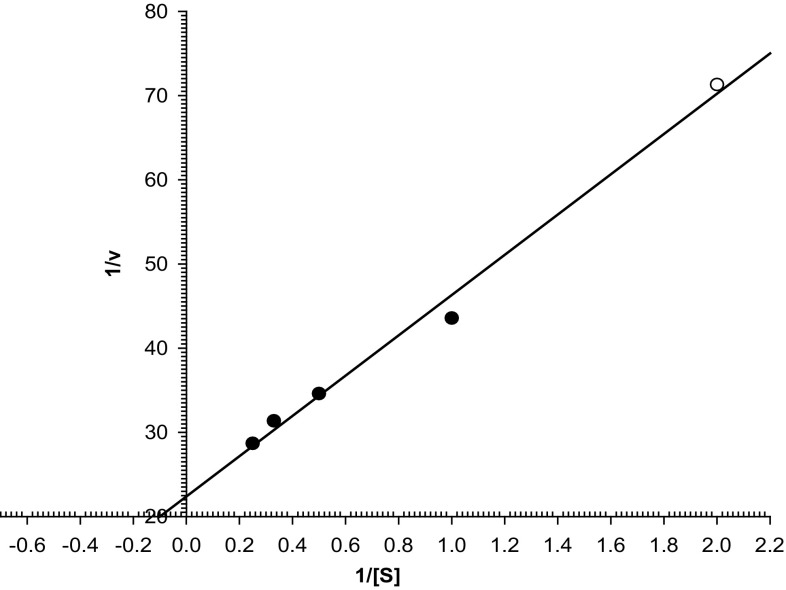
Lineweaver–Burk plot

The kinetic parameters for the CGTase catalyzed reaction were determined under standard assay conditions using starch as substrate. *K*_m_ and *V*_max_ values obtained for purified CGTase were 10 mg/ml and 146 µmol/mg min, respectively (Fig. 6).

The higher value of *K*_m_ indicates the lower affinity for the substrate and hence the reaction rate. The CGTase from *Pae. campinasensis* H69-3 has the K_m_ and *V*_max_ value of 1.69 mg/ml and 4.97 µmol/min mg, respectively (Alves-Prado et al. 2007). The *K*_m_ and *V*_max_ values were 0.48 mg/ml and 51.38 mg/ml min, respectively, for *Paenibacillus illinoisensis* ZY-08 CGTase (Lee et al. 2013). The values of *K*_m_ 14.3 g/l and *V*_max_ 106 U/mg were reported using potato starch as substrate for *Haloferax mediterranei* (Bautista et al. 2012). The *K*_m_ of 21.2 mg/ml and *V*_max_ of 7.4 µmol/mg min have been reported for the CGTase from *B. agaradhaerens* LC-3C (Martins and Hatti-Kaul 2002). The *K*_m_ and *V*_max_ values obtained were 0.15 mg/ml and 60.39 mg/ml min, respectively, for *Bacillus* sp. G1 CGTase (Sian et al. 2005). *K*_m_ 2.2 mg/ml and *V*_max_ 7.8 mg/ml min were reported for CGTase of *B. lehensis* (Elbaz et al. 2015).

## Conclusion

The present study describes the single step purification of cyclodextrin glucanotransferase using the starch adsorption method. The purified CGTase with 45.22 U/mg specific activity gave a single band in SDS-PAGE. To the best of our knowledge, this is the smallest molecular weight CGTase with 27.72 kDa reported so far. The purified enzyme works efficiently near neutral pH and at 60 °C temperature. The CGTase activity can be enhanced in the presence of Ca^+2^ and other metal ions. It remains active in several organic solvents with toluene as the best. The addition of PEG-6000 can increase the enzyme activity. These diverse properties of novel CGTase can be potentially exploited for industrial cyclodextrin production.
